# Habitat Radiomics Predict HPV Status in Oropharyngeal Cancer

**DOI:** 10.1002/cam4.71481

**Published:** 2025-12-21

**Authors:** Oya Altinok, Ghulam Rasool, Asim Waqas, Matthew B. Schabath, Albert Guvenis

**Affiliations:** ^1^ Department of Cancer Epidemiology H. Lee Moffitt Cancer Center & Research Institute Tampa Florida USA; ^2^ Institute of Biomedical Engineering Bogazici University Istanbul Turkiye; ^3^ Department of Machine Learning H. Lee Moffitt Cancer Center and Research Institute Tampa Florida USA

**Keywords:** biomarkers, habitat imaging, head and neck cancer, HPV status, imaging‐based algorithm, oropharyngeal cancer, tumor heterogeneity

## Abstract

**Objective:**

This study developed a habitat‐based radiomics classifier from CT images to predict HPV status in oropharyngeal cancer (OPC).

**Methods:**

We analyzed pretreatment CT scans from 192 OPC patients. Tumor habitats were generated using a two‐level unsupervised clustering approach, and radiomics features were calculated from both intratumoral and habitat‐defined subregions. HPV ground truth was based on p16 IHC, and the ROSE algorithm was used to address class imbalance (85% HPV‐positive). LASSO regression was used for feature selection. We developed and compared three separate models for statistical performance to predict HPV status: habitat radiomics classifier, intratumoral radiomics classifier, and combined radiomics classifier (habitats and intratumoral). Classifier performance was assessed using area under the receiver operating characteristic curve (AUCROC), and SHAP (SHapley Additive Explanations) was utilized to interpret features contributing to classifier predictions. Kaplan–Meier analysis was conducted to compare survival outcomes of HPV ground truth versus the radiomics classifier.

**Results:**

The habitat radiomics classifier significantly outperformed the intratumoral radiomics classifier, achieving AUCs of 0.970 (95% CI, 0.942–0.997) in the training cohort and 0.937 (95% CI, 0.843–1.00) in the test cohort. The combined radiomics classifier did not significantly improve performance. SHAP revealed that radiomic features of compact/spherical shape and uniform texture were associated with HPV‐positive tumors, reflecting lower morphologic and textural heterogeneity than HPV‐negative tumors. For the survival analysis, the HPV habitat radiomics classifier is indistinguishable from HPV ground truth in predicting overall survival (*p* = 0.52 for HPV‐negative ground truth vs. HPV‐negative classifier; *p* = 0.75 for HPV‐positive ground truth vs. HPV‐positive classifier).

**Conclusion:**

The habitat radiomics classifier provides an imaging‐based algorithm to predict HPV status in OPC by capturing spatial patterns of tumor heterogeneity that reflect biological differences between HPV‐positive and HPV‐negative tumors. This approach demonstrated strong performance, with additional support from SHAP‐based interpretability and Kaplan–Meier survival analysis suggesting its potential clinical relevance.

## 1 Introduction

The incidence rate of oropharyngeal cancer (OPC) is increasing both globally [1] and in the United States [2], attributed mainly to the rise in human papillomavirus (HPV)‐related disease [3]. Compared to HPV‐negative tumors, HPV‐positive tumors tend to be less heterogeneous and are often associated with better prognosis and response to treatment [4, 5, 6, 7]. OPC shows marked tumor heterogeneity, with differences between HPV‐positive and HPV‐negative tumors, further influenced by factors such as tobacco and alcohol use, oral hygiene, diet, and genetics [4, 8].

In the clinical setting, HPV status is determined using immunohistochemistry (IHC), DNA/RNA in situ hybridization (ISH), or DNA/RNA polymerase chain reaction (PCR) [9]. However, these techniques have several limitations. First, they are typically performed on a limited tissue sample, which may capture only part of a heterogeneous lesion and therefore may not fully represent the tumor's biological complexity. Second, these methods require specimens and cannot be performed rapidly. Thus, it is valuable to explore imaging‐based algorithms and rapid approaches for determining the HPV status of OPC patients.

As such, there is an emerging and critical need to develop imaging‐based algorithms and rapidly measured biomarkers for clinical decision support. Image‐ and radiomics‐based methods can rapidly identify tumor habitats from standard‐of‐care medical images and can capture data from the entire region of interest rather than a small portion of the biopsied tumor [10]. Thus, an image‐based classifier to predict HPV status is a novel strategy for capturing the biological characteristics of a cancer. Numerous studies have demonstrated the efficacy of radiomics approaches [11]. However, those studies primarily focused on the entire tumor (i.e., intratumor), potentially overlooking spatial variations within the tumor.

Tumor heterogeneity is the biological diversity at the genetic, epigenetic, proteomic, and phenotypic levels [12, 13, 14]. Given the important biological, radiological, and clinical differences between HPV‐positive and HPV‐negative OPC tumors [5, 15], we hypothesized that these two tumor types could be distinguished based on imaging features that reflect underlying heterogeneity. Although intratumor‐based radiomics provide valuable information, some heterogeneity information may still be missed. Habitat imaging is an emerging technique in radiomics analysis that can provide quantitative image‐based markers of tumor heterogeneity. Habitats partition a region of interest (e.g., tumor) into distinct, spatially diverse, and heterogeneous sub‐volumes [16]. Many researchers have started to incorporate habitat imaging into the field of radiomics, showcasing its superior performance compared to other methods (i.e., intratumor radiomics) [17]. To date, habitat‐based radiomics has been less explored in OPC, and one study has applied it to assess treatment response [18]. Yet, its potential for predicting HPV status remains unexplored. Therefore, exploring habitat image features related to HPV status that can reflect the underlying biological characteristics of the tumors is an important strategy.

The goal of this study was to develop an image‐based classifier using habitat‐based radiomics to predict HPV status in OPC patients using contrast‐enhanced CTs. The interpretability of the image‐based classifier was assessed by SHAP (SHapley Additive Explanations) to reveal which features contribute to the prediction. Since HPV status is highly prognostic, we also conducted head‐to‐head survival analysis comparing the HPV image‐based classifier versus HPV ground truth.

## 2 Methods

### 2.1 Patient Data

**FIGURE 1:**
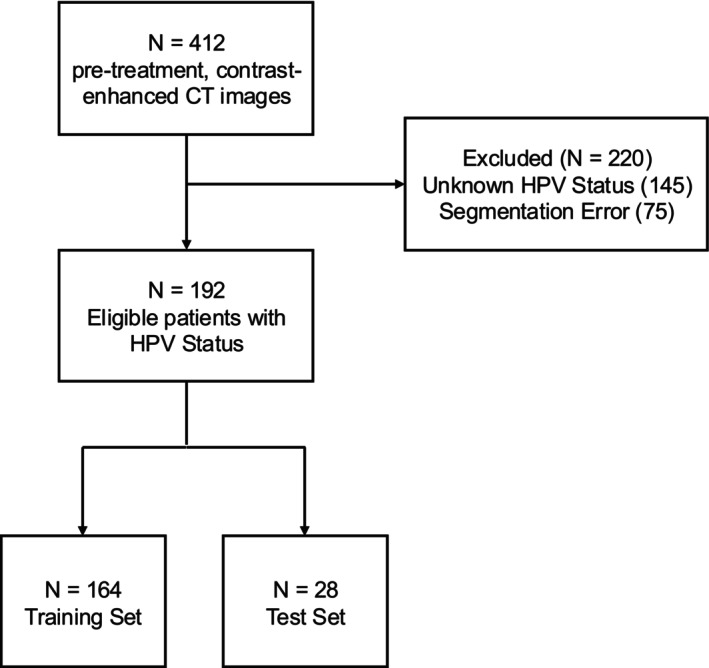
CONSORT diagram for patient inclusion. Patients who had unknown HPV status were excluded, and if there was a segmentation error (e.g., there was no RT struct, the file could not open, etc.). Among the 192 patients, 85% (*n* = 164) were assigned to the training set and 15% (*n* = 28) to the held‐out test set, using stratified splitting to preserve the HPV status distribution.

**FIGURE 2:**
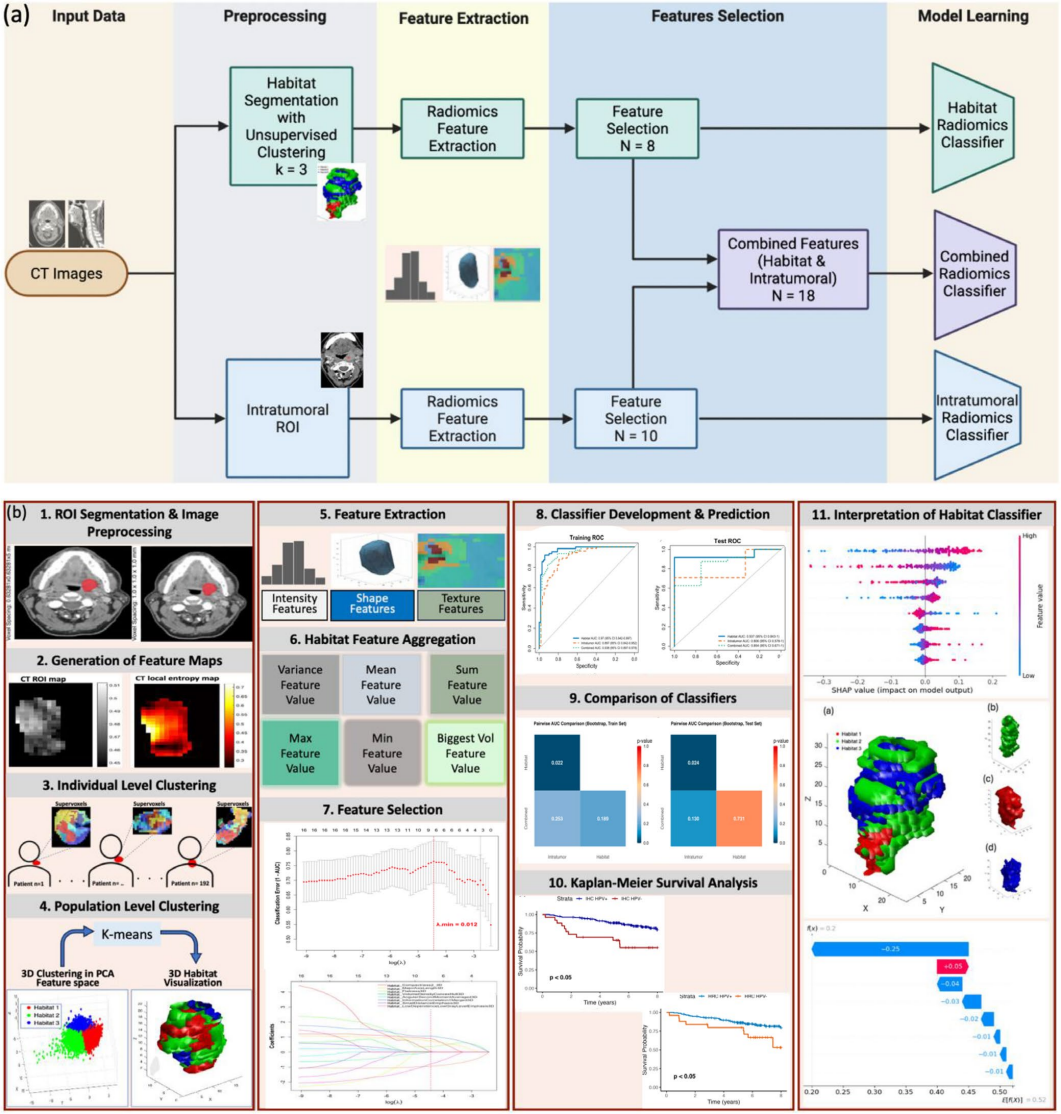
(a) Workflow diagram illustrating radiomic feature extraction and classification from contrast‐enhanced CT images. Unsupervised clustering was used to generate the habitat subregions from within the segmented tumor. Radiomic features were calculated from each habitat and the entire region of interest (intratumoral region). The habitat‐based classifiers were constructed by concatenating the selected features. Three separate classifiers were developed and compared for statistical performance to predict HPV status in OPC patients: Habitat‐based radiomics, intratumoral radiomics, and combined radiomics (habitats and intratumoral). (b) The radiomics workflow. The workflow used in the study includes: (1) Tumor segmentation and image preprocessing: CT images are resampled, and tumor masks are extracted. (2) Generation of feature maps: Feature maps such as entropy are computed from the CT images. (3) Individual‐level clustering: For each patient, supervoxels are created by grouping nearby voxels that have similar values in the feature maps from Step 2. (4) Population‐level clustering: The supervoxels from all patients are grouped based on their feature values from Step 3. This helps to find similar regions across patients. These groups are called “habitats” and may reflect shared tumor characteristics. (5) Radiomic feature extraction: Shape, intensity, and texture features are extracted from each habitat region. (6) Habitat‐level feature aggregation: Different strategies were tested to determine which habitat‐level feature set to use in the habitat radiomics classifier. Using the feature set from the habitat with the maximum (Max) feature value yielded the best performance and was used in the final classifier. (7) Feature selection: Key features are selected using LASSO logistic regression, which removes less important features by shrinking their coefficients to zero. The optimal level of shrinkage is chosen through cross‐validation. (8) Classifier development and prediction: Radiomics classifiers are trained and evaluated using the selected features. (9) Performance comparison: Classifier performance is compared across the habitat radiomics classifier, intratumoral radiomics classifier, and combined radiomics classifier. (10) Survival analysis: Kaplan–Meier curves compare survival based on predicted versus true HPV status. (11) Interpretation of the habitat classifier: SHAP beeswarm plots and SHAP waterfall plots, along with 3D visualizations of tumor habitats, are used to interpret the model's predictions.

The dataset used in this analysis was obtained from The Cancer Imaging Archive (TCIA) and consists of 192 patients diagnosed with OPC. The data include pre‐treatment, contrast‐enhanced CT images, having segmentation labels [19, 20, 21], and HPV status (p16 protein‐positive or negative). The gross primary tumor volume (GTVp), segmented by experts [20], was considered in the radiomic analysis and did not include nodal disease (LN) volumes. A CONSORT diagram illustrating patient inclusion is shown in Figure 1, and the overall study workflow is provided in Figure 2.

### 2.2 Image Preprocessing

All images and their corresponding segmentations for the region of interest (ROI) were resampled to a 1 mm^3^ isotropic voxel space. We employed cubic interpolation for CT images and nearest‐neighbor interpolation for segmentations. Z‐score normalization was then applied to standardize intensity values across patients. Image preprocessing was performed using MATLAB 2024b.

### 2.3 Habitat Generation

We adapted open‐source implementations from two prior studies (https://github.com/WenbingLv/Subregional‐Radiomics) and (https://github.com/WuLabMDA/Habitat‐Analysis) to generate the habitats from the ROI [22, 23]. The following steps were utilized: (1) generation of feature maps, (2) individual‐level clustering, and (3) population‐level clustering. The details of the steps are described in the following paragraphs.

For the generation of feature maps, in addition to the original voxel intensity on CT, CT local entropy maps were generated by computing the first‐order intensity entropy within a small neighborhood (9 x 9 x 9 neighborhood) for each CT voxel. This step allows us to measure intratumoral heterogeneity locally. This map measures the degree of chaos in a small neighborhood of each voxel. The CT and CT entropy images were fused by voxel‐wise addition to produce a composite image, which was then used for subsequent clustering.

For the individual‐level clustering, at the patient level, feature maps of each GTVp (primary tumor) were independently over‐segmented into numerous supervoxels by the SLIC (Simple Linear Iterative Clustering) algorithm [24], with squared Euclidean distance between voxel intensities as the similarity metric. Then, based on gray‐level frequency distribution from the histogram analysis, the first‐order features (*n* = 20) were extracted for individual supervoxels, including skewness, kurtosis, mean, median, first quartile, second quartile, interquartile range, standard deviation, variance, and energy calculated based on two different maps. Thus, each supervoxel was represented by 20 first‐order statistical features calculated separately from the CT and CT entropy images.

For the population‐level clustering, we pooled all patients' supervoxels' features in the tumor and performed the K‐means clustering method. Similar habitats are generated among all supervoxels using the K‐means clustering method. In accordance with earlier studies that explored cluster numbers in the range of 3–10 for similar data types [25, 26], we evaluated clustering performance using the Calinski–Harabasz (CH) index [27] to determine the optimal number of clusters. The optimal k was selected based on the maximum CH score, which indicates the most distinct and compact clustering structure of the tumor habitats.

The Supplemental Data provides additional details of habitat generation, including mathematical formulations of SLIC, K‐means, and the CH index.

### 2.4 Feature Extraction

Radiomic features in this study were extracted from distinct tumor habitats and categorized into three groups: (I) shape, (II) intensity, and (III) texture features. All features were extracted using MATLAB 2024b's radiomics toolbox, which adheres to the Image Biomarker Standardization Initiative (IBSI) guidelines [28].

### 2.5 Habitat Feature Aggregation Strategies

To integrate information across tumor habitats, multiple feature aggregation strategies were defined, including variance, maximum, minimum, mean, and summed feature values, as well as selection based on the habitat with the largest volume (Table S1). These strategies were evaluated within the nested cross‐validation framework described in the following Training and Test Splitting section, where a separate classifier was trained and assessed for each strategy. Among all the evaluated strategies, the maximum feature value approach, where the highest value of each radiomic feature across the three habitats was selected, achieved the highest AUC during cross‐validation. Therefore, this strategy was employed in the final habitat radiomics classifier.

### 2.6 Training and Test Datasets

We applied 7‐fold nested cross‐validation to develop and evaluate the classifiers. In each outer fold, six folds of the data were used for classifier development, while one fold was kept strictly for testing. Within the training set, feature selection and hyperparameter tuning were performed. Stratified splitting was applied to preserve the original distribution of HPV status. Notably, the test data remained completely unseen during feature selection, data balancing, and model training, ensuring that no data leakage occurred.

### 2.7 Feature Selection

A multi‐step feature preprocessing and selection pipeline was applied before classifier development. First, min‐max normalization was applied to scale all radiomic features in the training set to the range between −1 and 1. The same scaling parameters were then used to normalize the test set. Next, a two‐sample t‐test was conducted on the training set to identify features significantly associated with the outcome (*p* < 0.05). Highly correlated features (Pearson correlation |r| > 0.9) were then filtered by retaining the feature with the smaller *p*‐value from the t‐test. Finally, the Least Absolute Shrinkage and Selection Operator (LASSO) logistic regression was employed to reduce dimensionality further. The optimal regularization parameter (λ) was determined using 10‐fold cross‐validation, and features with non‐zero coefficients at this value were retained for classifier development. The same feature selection strategy was independently applied to both habitat‐based and intratumoral radiomic features. For the combined radiomics classifier, we constructed the feature set by merging the features that were individually selected during the feature selection process for the habitat‐based and intratumoral radiomics classifiers. To address class imbalance in the training cohort, synthetic data balancing was performed using the ROSE algorithm [29].

### 2.8 Classifier Development

Three classifiers were developed to predict HPV status: (1) a habitat radiomics classifier based on eight features derived from habitat‐based analysis, (2) an intratumoral radiomics classifier using ten selected features from whole‐tumor analysis, and (3) a combined radiomics classifier that combined the final selected features from the habitat radiomics classifier and intratumoral radiomics classifier. All classifiers were trained using a support vector machine (SVM) with a radial basis function (RBF) kernel. Each classifier underwent its own nested cross‐validation framework to ensure fair and unbiased evaluation. A 7‐fold nested cross‐validation was used to tune hyperparameters and assess performance. In each fold, the model was trained using the selected features, and the best‐performing hyperparameters were identified through 3‐fold cross‐validation within the training data, based on the area under the ROC curve (AUC). The final model was then evaluated on the corresponding held‐out test set. After completing all folds, the model from the fold with the highest test AUC was selected as the final classifier.

### 2.9 Classifier Performance and Survival Analysis

Classifier performance was evaluated using the area under the curve (AUC). To compare AUCs between the classifiers, bootstrap tests with 1000 repeats were employed. Additionally, the SHAP method was employed to interpret the best‐performing classifier, using both beeswarm and waterfall plots to visualize feature contributions [30].

Kaplan–Meier (KM) survival analysis was performed using both the predicted HPV status from the best‐performing classifier and the true HPV status. Patients were stratified into HPV‐positive and HPV‐negative groups, and survival differences were compared using the log‐rank test. A *p*‐value < 0.05 was considered statistically significant.

### 2.10 Statistical Analysis

Continuous variables were presented as mean ± standard deviation (SD) or median with interquartile range (IQR), as appropriate. Categorical variables were compared using the Chi‐square test or Fisher's exact test, as applicable. All *p*‐values were two‐sided, and a *p* < 0.05 was considered statistically significant. Analyses were performed in R (version 4.4.2).

## 3 Results

### 3.1 Patient Characteristics

A total of 192 patients were included. Stratified splitting assigned 164 to the training set and 28 to the test set, preserving HPV status. Patient characteristics were similar across sets. Detailed patient characteristics are provided in Table 1 and Table S4.

**TABLE 1:** Comparison of patient characteristics between training and test sets.

Patient Characteristics	All Patients (*N* = 192)	Training (*N* = 164)	Test (*N* = 28)	*p*
Mean Age [years ± SD]	58.13 (9.25)	58.29 (9.12)	57.21 (10.14)	0.572
Survival Time [median IQR]	6.22 [4.91, 7.67]	6.21 [4.91, 7.53]	6.94 [5.28, 8.64]	0.108
Gender (%)				0.460
Male	157 (81.8)	136 (82.9)	21 (75.0)	
Female	35 (18.2)	28 (17.1)	7 (25.0)	
HPV Status (%)				1.000
Positive	166 (86.5)	142 (86.6)	24 (85.7)	
Negative	26 (13.5)	22 (13.4)	4 (14.3)	
Smoking Status (%)				0.962
Current	45 (23.4)	39 (23.8)	6 (21.4)	
Former	77 (40.1)	65 (39.6)	12 (42.9)	
Never	69 (35.9)	59 (36.0)	10 (35.7)	
NA	1 (0.5)	1 (0.6)	0 (0.0)	
Vital Status (%)				0.133
Alive	155 (80.7)	129 (78.7)	26 (92.9)	
Dead	37 (19.3)	35 (21.3)	2 (7.1)	
T Category (%)				0.534
1	44 (22.9)	35 (21.3)	9 (32.1)	
2	78 (40.6)	68 (41.5)	10 (35.7)	
3	40 (20.8)	36 (22.0)	4 (14.3)	
4	30 (15.6)	25 (15.2)	5 (17.9)	
N Category (%)				0.402
0	20 (10.4)	17 (10.4)	3 (10.7)	
1	28 (14.6)	25 (15.2)	3 (10.7)	
2	139 (72.4)	119 (72.6)	20 (71.4)	
3	5 (2.6)	3 (1.8)	2 (7.1)	
AJCC Stage 7th Edition (%)				0.297
I	2 (1.0)	1 (0.6)	1 (3.6)	
II	7 (3.6)	7 (4.3)	0 (0.0)	
III	36 (18.8)	32 (19.5)	4 (14.3)	
IV	147 (76.6)	124 (75.6)	23 (82.1)	

### 3.2 Habitat Clusters

**FIGURE 3:**
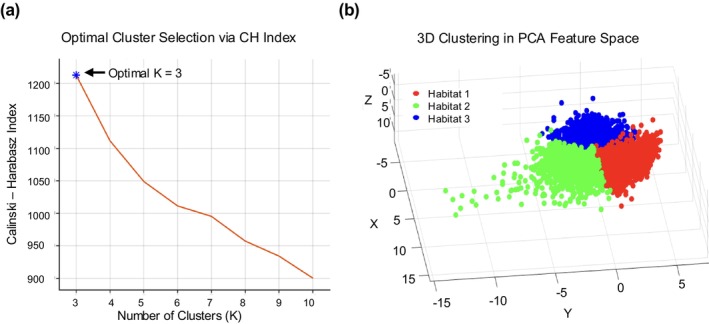
(a) Assessment of clustering performance using the Calinski–Harabasz (CH) index. (b) Visualization of habitat features segmented into three clusters. CH Index: Quantifies the ratio of between‐cluster dispersion to within‐cluster compactness, with higher values reflecting superior clustering quality.

The optimal number of clusters for habitat generation was determined to be K = 3, based on the maximum value of the Calinski–Harabasz (CH) index (Figure 3a). Clustered supervoxel features were visualized in a three‐dimensional PCA‐transformed feature space, revealing clear separation among the three habitat groups (Figure 3b). Each color represents a distinct tumor habitat, with Habitat 1 (red), Habitat 2 (green), and Habitat 3 (blue), capturing spatially distinct and heterogeneous regions within the tumor microenvironment.

### 3.3 Selected Features

**FIGURE 4:**
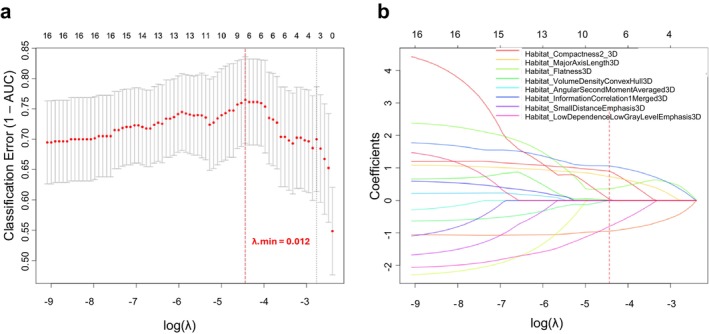
Feature selection using the least absolute shrinkage and selection operator (LASSO) logistic regression. (a) Tuning parameter (λ) selection in the LASSO. Classification error (1—AUC) was plotted against log(λ). The optimal value of λ was selected using 10‐fold cross‐validation and is indicated by the vertical red dashed line. (b) LASSO coefficient profiles of the features. Each colored line represents the trajectory of a specific feature's coefficient. The vertical red dashed line marks the selected λ value, where 8 features had nonzero coefficients.

Habitat‐level radiomic features were extracted by calculating the same 208 features for each of the three tumor subregions (208 × 3), grouped into shape (23), first‐order intensity (49), and texture (136) categories. Additionally, radiomic features were independently extracted from the whole tumor, resulting in 208 features across the same three categories. Feature selection was performed using the LASSO logistic regression method, with optimal lambda (λ) values determined separately for each classifier to minimize classification error. For the habitat radiomics classifier, the optimal λ value was 0.012, resulting in the selection of 8 features with non‐zero coefficients. For the intratumoral radiomics classifier, the optimal λ was 0.0082, leading to the retention of 10 features. The tuning process and corresponding coefficient profiles for the habitat radiomics classifier are illustrated in Figure 4a–b. The LASSO feature selection results for the intratumoral radiomics classifier are presented in Figure S1. The final sets of selected features for all three classifiers are summarized in Table S3.

### 3.4 Performance and Comparisons of Radiomics Classifiers

**FIGURE 5:**
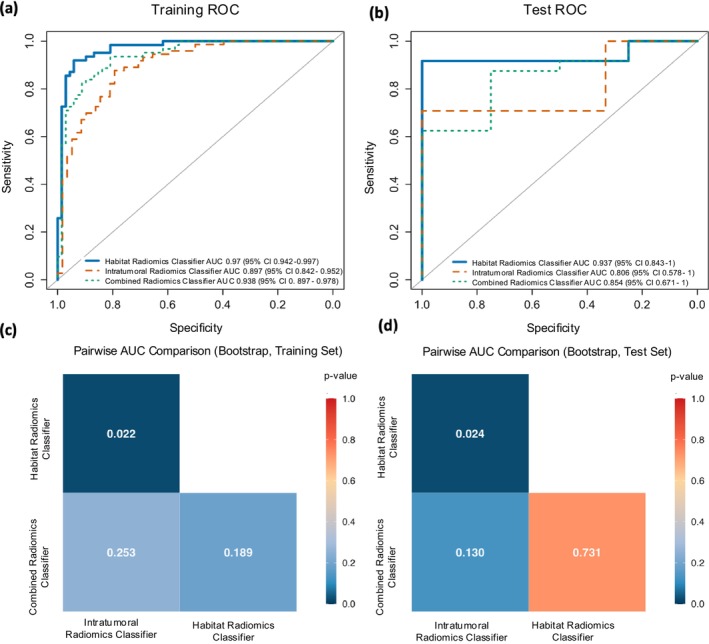
Receiver operating characteristic (ROC) curves and pairwise AUC comparisons of radiomics classifiers for HPV status prediction. (a) ROC curves for the training set and (b) for the test set, comparing three classifiers: Habitat radiomics (solid blue), intratumoral radiomics (dashed orange), and combined radiomics (dotted green). Pairwise comparison of AUCs using bootstrapping in the training set (c) and test set (d): Comparisons between Habitat radiomics classifier vs. Intratumoral radiomics classifier, Intratumoral radiomics classifier vs. Combined radiomics classifier, and Combined radiomics classifier vs. Habitat radiomics classifier.

Classifier performance comparisons are presented in Table 2, and the AUC curves of all classifiers are shown in Figure 5. In both the training and test sets, the habitat radiomics classifier achieved higher AUCs than the intratumoral radiomics classifier, with values of 0.970 (95% CI: 0.942–0.997) vs. 0.897 (95% CI: 0.842–0.952) in the training set and 0.937 (95% CI: 0.843–1.000) vs. 0.806 (95% CI: 0.578–1.000) in the test set. Pairwise AUC comparisons using bootstrap resampling (Figure 5c–d) confirmed that the habitat radiomics classifier significantly outperformed the intratumoral radiomics classifier in both the training set (*p* = 0.022) and the test set (*p* = 0.024). The combined radiomics classifier achieved AUCs of 0.938 (95% CI: 0.897–0.978) in the training set and 0.854 (95% CI: 0.671–1.000) in the test set. The combined method did not result in a statistically significant improvement (Figure 5c–d).

**TABLE 2:** Performance evaluation of all classifiers for the training and test datasets.

Datasets	Classifiers	AUC (95% CI)	Accuracy (95% CI)	Sensitivity (95% CI)	Specificity (95% CI)
Training	Habitat Radiomics Classifier	0.970 (0.942–0.997)	0.908 (0.846–0.946)	0.956 (0.747–0.922)	0.855 (0.878–0.985)
	Combined Radiomics Classifier	0.938 (0.897–0.978)	0.862 (0.792–0.911)	0.897 (0.808–0.960)	0.823 (0.750–0.914)
	Intratumoral Radiomics Classifier	0.897 (0.842–0.978)	0.832 (0.759–0.886)	0.759 (0.700–0.874)	0.890 (0.798–0.960)
Test	Habitat Radiomics Classifier	0.937 (0.843–1.00)	0.821 (0.644–0.921)	0.792 (0.595–0.908)	1.000 (0.701–1.000)
	Combined Radiomics Classifier	0.854 (0.671–1.000)	0.821 (0.644–0.921)	0.875 (0.698–0.963)	0.500 (0.376–0.964)
	Intratumoral Radiomics Classifier	0.806 (0.578–1.00)	0.704 (0.515–0.841)	0.708 (0.494–0.877)	0.667 (0.607–0.990)

In the training set, the habitat radiomics classifier achieved an accuracy of 0.908 (95% CI: 0.846–0.946), a sensitivity of 0.956 (95% CI: 0.747–0.922), and a specificity of 0.855 (95% CI: 0.878–0.985). In the test set, its accuracy was 0.821 (95% CI: 0.644–0.921), sensitivity was 0.792 (95% CI: 0.595–0.908), and specificity was 1.000 (95% CI: 0.701–1.000). Other classification metrics, including precision, recall, and F1‐score for both positive and negative classes, are reported in Table S2 for all three classifiers on both the training and test datasets.

### 3.5 Multilevel Interpretation of Habitat Radiomics Classifier

In this section, we provide a multilevel interpretation of the habitat radiomics classifier to facilitate understanding of its predictive behavior. This includes global feature importance interpreted by SHAP analysis, distributional comparisons of discriminative habitat features, and patient‐specific feature attributions presented with SHAP.

#### 3.5.1 Global Feature Importance Interpreted by SHAP Analysis

**FIGURE 6:**
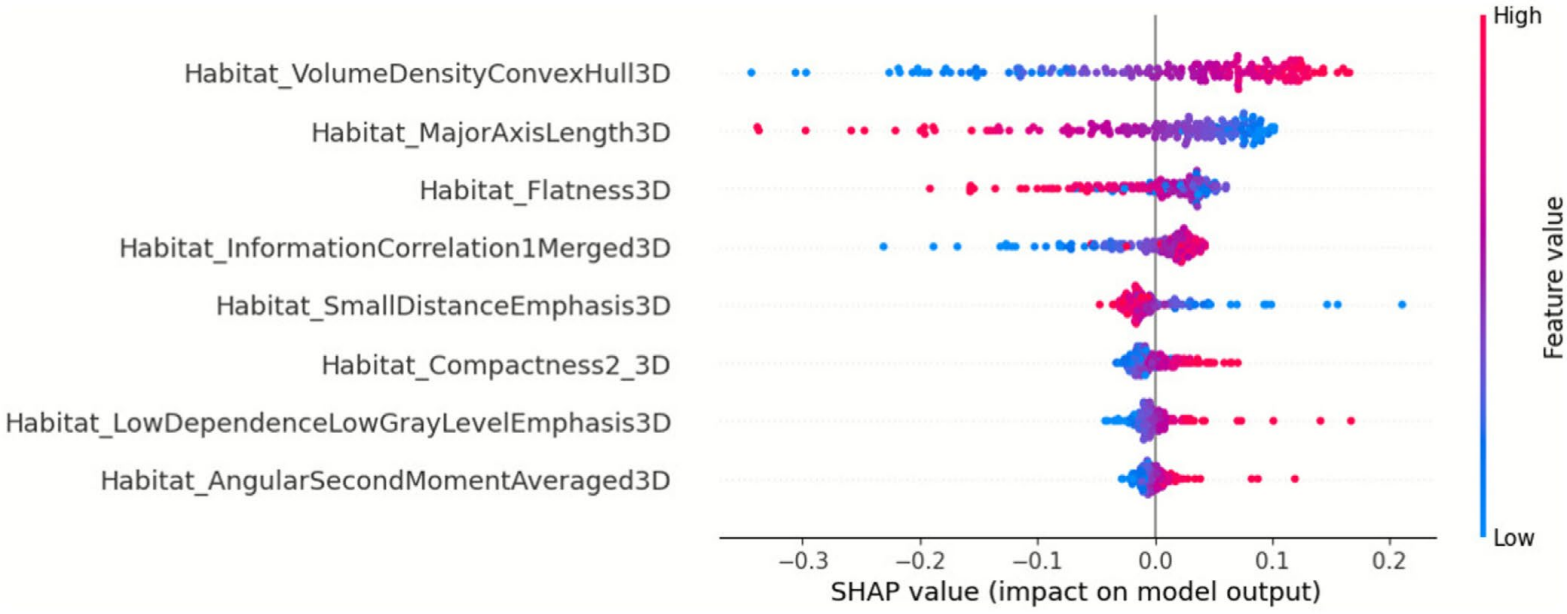
SHAP beeswarm plot for the habitat radiomics classifier, illustrating the impact of habitat‐derived radiomic features on HPV status prediction in the training dataset. Each point represents a patient, with the SHAP value on the x‐axis indicating the contribution of a specific feature to the classifier's output. Positive SHAP values correspond to an increased likelihood of an HPV‐positive prediction. Red data points represent a higher absolute value of the feature, while blue data points represent a lower value. Among the top contributing features: (1) higher values of Volume Density Convex Hull 3D increased the likelihood of an HPV‐positive prediction; (2) lower values of Major Axis Length 3D values were linked to HPV‐positive cases; (3) Lower Flatness 3D values (i.e., less flat tumors) also contributed to HPV‐positive classification.

Figure 6 presents the SHAP beeswarm plot for the best‐performing model (habitat classifier), which summarizes the global impact of each feature on the classifier's predictions across all patients. Each point represents a single patient, and the color indicates the feature value (red = high, blue = low). Features are ranked by their average contribution to the prediction, helping to identify which radiomic features are most influential in HPV status classification. Among the features, VolumeDensityConvexHull3D had the highest impact, with higher values associated with HPV‐positive predictions. Lower values of MajorAxisLength3D, Flatness3D, and Compactness2_3D were also linked to HPV‐positive cases, indicating more compact and spherical tumor shapes.

For texture‐based features, higher values of InformationCorrelation1Merged3D were associated with HPV‐positive predictions. In contrast, lower values of SmallDistanceEmphasis3D and LowDependenceLowGrayLevelEmphasis3D were linked to HPV‐positive status, suggesting smoother texture patterns. Additionally, AngularSecondMomentAveraged3D was lower in HPV‐negative tumors, reflecting greater texture uniformity in HPV‐positive cases. SHAP analysis results for the intratumoral radiomics classifier are presented in Supplementary Figure S2.

#### 3.5.2 Box Plot Visualization of Discriminative Habitat Features

**FIGURE 7:**
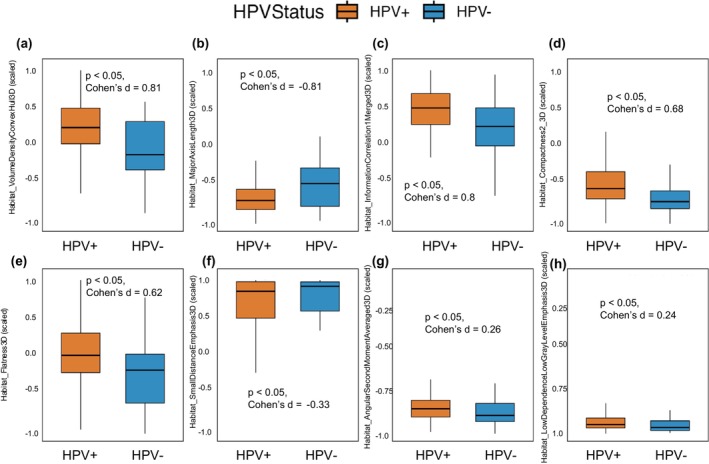
Box plots of radiomic features selected for the habitat radiomics classifier. Each feature is shown for HPV‐positive (HPV+, orange) and HPV‐negative (HPV−, blue) patient groups. All features were scaled to the [−1, 1] range. median + IQR (interquartile range). *p*‐values from independent t‐tests and corresponding Cohen's d effect sizes are displayed above each panel (a–h). The sign of Cohen's d reflects the direction of the group difference: Positive values indicate higher feature values in HPV+ patients, and negative values indicate higher values in HPV− patients. Mean+/− std. dev.

We further calculated the distributions of each selected feature from the habitat classifier to provide complementary value. Figure 7 displays the distribution of selected habitat radiomic features across HPV‐positive and HPV‐negative patients. Similarly, for the intratumoral radiomics classifier, the distribution of selected features is provided in Supplementary Figure S3.

#### 3.5.3 Patient‐Specific Feature Attribution With SHAP

**FIGURE 8:**
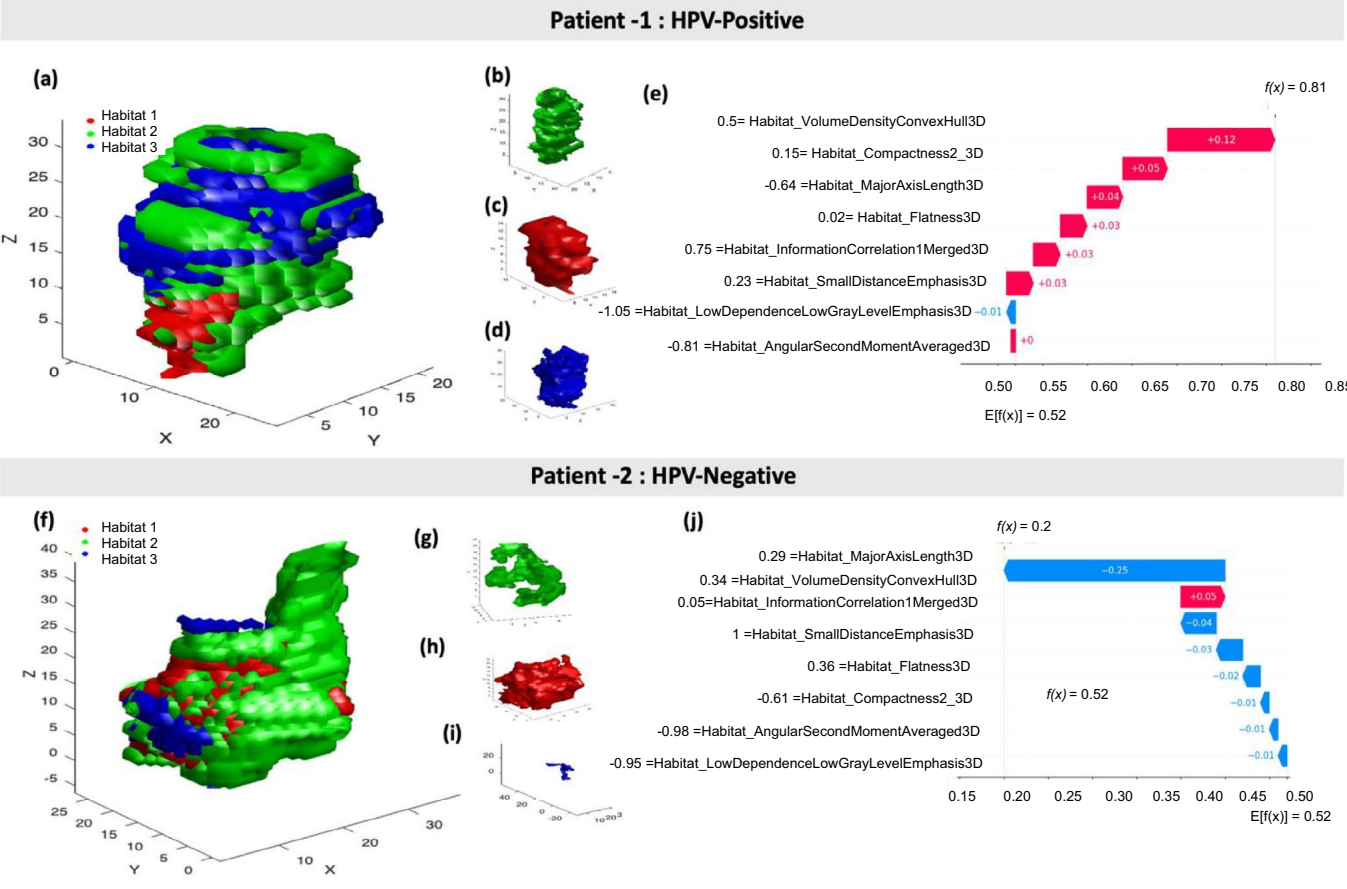
Habitat‐based visualization and SHAP interpretation for two example patients. (a–e) Patient 1 (HPV‐Positive): (a) Tumor with overlaid habitats (red: Habitat 1, green: Habitat 2, blue: Habitat 3), (b–d) individual habitat shapes, (e) SHAP waterfall plot showing how radiomic features derived from the tumor habitats contribute to the prediction of HPV‐positive status. (f–j) Patient 2 (HPV‐Negative): (f) Tumor with overlaid habitats, (g–i) individual habitat shapes, (j) SHAP waterfall plot showing feature contributions leading to an HPV‐negative prediction. SHAP waterfall plot explains the prediction for a single patient by showing how each feature affects the final classifier output. The plots begin at the baseline value E[f(x)], which represents the average classifier output across all training samples, and illustrate how individual features incrementally push the prediction higher or lower to reach the final score f(x). Each bar corresponds to one feature, with the numerical value next to it indicating the SHAP value, that is, the strength and direction of that feature's effect on the prediction. Pink bars represent features with positive SHAP values, pushing the prediction toward the HPV‐positive class. Blue bars represent features with negative SHAP values, pulling the prediction toward the HPV‐negative class. The final prediction score f(x) (e.g., 0.81 for patient 1 and 0.20 for patient 2) represents the classifier's output probability for the HPV‐positive class after accounting for all feature effects. Higher values indicate stronger confidence in HPV positivity, while lower values reflect a prediction toward the HPV‐negative class.

Figure 8 illustrates two example patients to demonstrate how the habitat radiomics classifier uses radiomics features to make individualized predictions. Panels 8 (a–d) and (f–i) display 3D visualizations of tumor habitats for an HPV‐positive and an HPV‐negative patient, respectively. SHAP waterfall plots (Figure 8e,j) provide patient‐level explanations by showing how each radiomic feature contributed to the prediction. In these plots, each bar represents the contribution of a single feature to the classifier's prediction. Pink (positive) bars indicate features that increase the prediction score, while blue (negative) bars indicate features that decrease it. The plot starts from the base value (i.e., the average classifier output across all patients) and moves step by step toward the final prediction, offering a top‐down view of how the classifier arrives at its decision for each patient.

For the HPV‐positive patient (Figure 8e), the classifier's prediction score increased from the base value of 0.52 to a final value of 0.81. The most influential features were VolumeDensityConvexHull3D (+0.12), Compactness2_3D (+0.05), and MajorAxisLength3D (+0.04), all of which pushed the prediction toward the HPV‐positive class. Additional features contributing positively included Flatness3D, InformationCorrelation1Merged3D, and SmallDistanceEmphasis3D, each adding approximately +0.03 to the prediction score. Only one feature, LowDependenceLowGrayLevelEmphasis3D, had a slight negative effect (−0.01), and AngularSecondMomentAveraged3D had a negligible impact. Altogether, this feature pattern strongly supported the classification as HPV‐positive.

For the HPV‐negative patient (Figure 8j), the classifier's prediction score decreased from the base value of 0.52 to a final value of 0.20. The largest negative contribution came from MajorAxisLength3D (−0.25), followed by InformationCorrelation1Merged3D (−0.04) and SmallDistanceEmphasis3D (−0.03), which collectively pushed the prediction toward the negative class. Other features, such as Flatness3D, Compactness2_3D, AngularSecondMomentAveraged3D, and LowDependenceLowGrayLevelEmphasis3D, also had small negative effects. Only one feature, VolumeDensityConvexHull3D, had a slight positive impact (+0.05) but was not sufficient to reverse the overall prediction. Altogether, this feature pattern strongly supported the classification as HPV‐negative.

### 3.6 Survival Analysis Based on HPV Status

**FIGURE 9:**
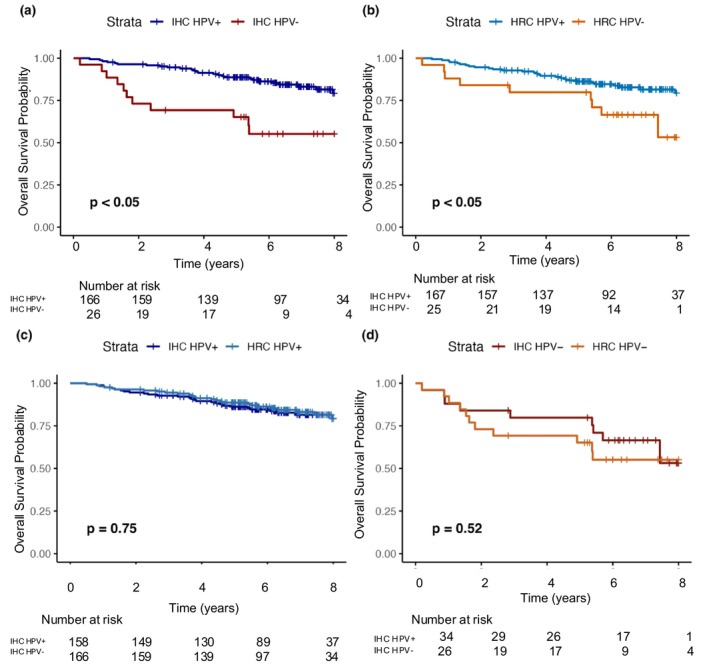
Kaplan–Meier overall survival curves. (a) Stratified by HPV status using immunohistochemistry (IHC) ground truth labels. (b) Stratified by predicted HPV status using the habitat radiomic classifier (HRC). (c) Comparison of survival for HPV‐positive patients classified by HRC vs. IHC. (d) Comparison of survival for HPV‐negative patients classified by HRC vs. IHC. HRC: Habitat Radiomics Classifier; IHC: Immunohistochemistry.

For the survival analyses (Figure 9), HPV‐positive patients (Figure 9a,b) exhibited significantly improved overall survival. Further, the HPV habitat‐radiomics classifier is indistinguishable from HPV ground truth in predicting overall survival (*p* = 0.524 for HPV‐negative ground truth vs. HPV‐negative classifier; *p* = 0.754 for HPV‐positive ground truth vs. HPV‐positive classifier).

## 4 Discussion

In this study, a comprehensive analytical approach was deployed to develop an image‐based classifier to predict HPV status from CT images. In this approach, we developed and compared the statistical performance of habitat‐based radiomics, intratumoral radiomics, and combined radiomics (habitats and intratumoral). A key innovation is the incorporation of habitat‐level radiomics to capture spatial heterogeneity and the use of SHAP (SHapley Additive Explanations) to enhance the interpretability of selected features. To our knowledge, this is the first study to apply habitat‐based radiomics for HPV prediction in OPC. Notably, the HPV habitat radiomics classifier achieved superior predictive performance compared to the intratumoral radiomics classifier. Moreover, underscoring its potential clinical utility, the HPV habitat radiomics classifier mirrored the differences in overall survival for HPV+ vs. HPV‐ compared to the ground‐truth HPV status.

Habitat‐based radiomics subdivides the tumor volume into distinct subregions, or habitats, based on image heterogeneity such as variations in texture or intensity [31]. These subregions are hypothesized to reflect underlying biological differences and have been used to predict several cancer‐relevant biomarkers, including *EGFR* mutation and nodal status in lung cancer [26, 32], HER2 status in breast cancer [33], and Ki‐67 expression in laryngeal cancer [34]. Despite growing evidence supporting this approach, its application to oropharyngeal cancer remains rare, with only one previous study using habitat analysis for response evaluation [18]. In contrast to conventional intratumoral radiomics, which average features over the entire tumor volume, it may mask phenotypic variation. Thus, the more pronounced radiomic differences observed between HPV‐positive and HPV‐negative tumors in our study may be attributed to the habitat‐based approach's enhanced ability to preserve and extract spatially resolved tumor phenotypes. This is further supported by the significantly higher AUC achieved by the habitat radiomics classifier compared to the intratumoral radiomics classifier. Overall, these findings suggest that habitat‐based radiomics not only improves classification performance but also enables more biologically meaningful interpretations of tumor heterogeneity and subtype differences.

The habitat radiomics classifier outperformed the intramural radiomics classifier in both the training cohort (AUC 0.970 vs. 0.897, respectively) and in the test cohort (AUC 0.937 vs. 0.806, respectively). This finding is consistent with previous studies reporting superior performance of habitat‐based radiomic classifiers over intratumoral classifiers [26, 34]. The combined radiomics classifier that integrated features from both habitat radiomics classifier and intratumoral radiomics classifier did not yield a statistically significant improvement. These findings raise the possibility that habitat‐derived features captured most of the prediction capacity. When compared with prior radiomics studies using CT imaging to predict HPV status, which reported AUCs ranging from 0.70 to 0.92 [11], it is notable that these earlier approaches predominantly relied on whole‐tumor features and did not incorporate habitat‐level segmentation or interpretable modeling strategies.

The habitat radiomics classifier is comprised of 4 shape‐ and volume‐based features and 4 texture‐based features. Based on SHAP analysis, “Volume Density Convex Hull 3D” emerged as the top contributor. Also known as “solidity” [35], this feature quantifies the compactness and geometric regularity of a tumor, and HPV‐positive tumors demonstrated higher solidity, suggesting more compact and smooth morphologies, while HPV‐negative tumors displayed irregular and spiculated contours. This finding aligns with Cantrell et al. [5], who observed that HPV‐negative tumors often exhibit ill‐defined borders. Other shape‐based features also supported these distinctions. HPV‐positive tumors showed lower values for “Major Axis Length 3D” and “Flatness 3D”, along with higher values for “Compactness2 3D,” indicating a tendency toward more spherical and homogeneous shapes. These results are consistent with prior whole‐tumor radiomic studies [36, 37, 38, 39], which reported that HPV‐positive tumors are typically more geometrically regular than their HPV‐negative counterparts. Texture‐based metrics further contributed to the predictive model. Higher values of “Information Correlation1 Merged 3D” were associated with HPV‐positive cases, suggesting more organized and homogeneous gray‐level patterns [40].

Notably, “Habitat Small Distance Emphasis 3D,” which derived from the Gray Level Distance Zone Matrix (GLDZM), ranked fifth in SHAP importance. This feature measures the proximity of similar‐intensity zones to the habitat boundary [41]. HPV‐positive tumors exhibited lower values, indicating centrally clustered homogeneous zones, whereas HPV‐negative tumors showed higher values, reflecting spatially dispersed textural patterns near the boundaries. Interestingly, this separation was only observable at the habitat level, not when the feature was calculated from whole‐tumor volumes, underscoring the utility of habitat segmentation in capturing localized heterogeneity. To our knowledge, this feature has not been reported in previous HPV‐focused radiomics studies. Lastly, the two lowest‐ranking SHAP features, “Low Dependence Low Gray Level Emphasis 3D” and “Angular Second Moment Averaged 3D,” were both higher in HPV‐positive tumors. These features also suggest greater textural uniformity, in line with findings by Song et al. [42], who reported lower intensity variation in HPV‐positive tumors. Although our study did not directly assess genomic pathways, the morphological (e.g., compactness, axis length) and textural (e.g., homogeneity) differences we found between HPV‐positive and HPV‐negative tumors are consistent with Zhu et al. [43], who linked similar imaging phenotypes to genomic mechanisms, reporting that HPV‐positive tumors tended to be more regular and homogeneous, whereas HPV‐negative tumors were larger, more irregular, and genomically aggressive.

A recent single‐cell RNA sequencing study revealed molecular heterogeneity between and within HPV‐positive and HPV‐negative OPC. Notably, distinct malignant cell populations were identified within HPV‐positive tumors, characterized by either detectable (HPVon) or undetectable (HPVoff) HPV gene expression, reflecting divergent transcriptional states [44, 45]. A habitat framework could be deployed to delineate regions that phenotypically resemble the HPVon state. Conversely, in HPV‐negative tumors, which display greater spatial and molecular heterogeneity [46], the subdivision of the intratumoral volume into distinct radiomic habitats may have provided a more nuanced representation of this complexity. Nevertheless, further research is needed integrating habitat radiomics with spatial transcriptomics to identify potential radiomic–genomic interplay.

Beyond classification performance, survival analyses further validated the clinical utility of the habitat‐based radiomics classifier. The predicted HPV‐positive and HPV‐negative groups showed significantly different survival outcomes (*p* < 0.05), closely mirroring the separation based on true HPV status (*p* < 0.05). Furthermore, Kaplan–Meier comparisons between predicted and actual groups revealed no significant differences (*p* = 0.52 for HPV‐negative; *p* = 0.75 for HPV‐positive). These results suggest that the survival outcomes of predicted HPV groups are indistinguishable from those based on molecular testing.

We do note that this study has some limitations. The sample size is relatively limited, but we used nested cross‐validation to provide a reliable evaluation. Class imbalance was also present, and we addressed it using ROSE to balance the training set. Additionally, we did not have an external validation dataset. Nonetheless, the rigorous analytical approach can be deployed in future studies that include larger and more diverse cohorts to ensure generalizability and support its potential clinical application. Another limitation is regarding KM: even though a high AUC was achieved for predicting HPV status and there were no statistically significant differences between the ground truth and the HRC classifier for predicting overall survival, we do acknowledge that there were some false positives (*N* = 21) and false negatives (*N* = 20), as well as true positives (*N* = 146) and true negatives (*N* = 5). We also acknowledge that p16 immunohistochemistry, used as a surrogate for HPV status in this dataset, may not perfectly correspond to HPV DNA results (e.g., [47]). This discordance could reflect biological heterogeneity among oropharyngeal tumors; thus, further studies integrating molecular HPV testing with imaging analyses are warranted.

## 5 Conclusion

This study developed and evaluated three pretreatment contrast‐enhanced CT‐based radiomic classifiers (habitat‐based, intratumoral, and combined) to predict HPV status in oropharyngeal cancer (OPC) patients. Among the classifiers tested, the habitat radiomic classifier outperformed both intratumoral and combined classifiers. By capturing spatial heterogeneity through tumor habitat analysis, the classifier identified key imaging features associated with HPV status. SHAP analysis further revealed that more compact shapes and uniform textures were strongly associated with HPV‐positive tumors. Moreover, Kaplan–Meier survival analysis supported the clinical relevance of the model's predictions. Overall, the habitat radiomics classifier offers a promising imaging algorithm for assessing HPV‐related heterogeneity in OPC.

## Author Contributions

**Oya Altinok oyaltinok:** conceptualization, methodology, writing – original draft, software, formal analysis, data curation, visualization. **Ghulam Rasool:** writing – review and editing. **Asim Waqas:** writing – review and editing, visualization. **Matthew B. Schabath:** writing – review and editing, supervision, methodology. **Albert Guvenis:** supervision, conceptualization, writing – review and editing, methodology.

## Ethics Statement

The authors have nothing to report.

## Conflicts of Interest

G.R. reports consulting fees from Impact Business Information Solutions (IBIS) Inc., Princeton, NJ 08542, USA. M.B.S. serves as an Associate Editor for Cancer Medicine.

## Supporting information


**Data S1:** Supplementary Figures and Tables

## Data Availability

The datasets generated and analyzed during the current study are available in the TCIA repository (https://www.cancerimagingarchive.net).

## References

[1] Z. S. Zumsteg , M. Luu , P. S. Rosenberg , et al., “Global Epidemiologic Patterns of Oropharyngeal Cancer Incidence Trends,” JNCI Journal of the National Cancer Institute 115, no. 12 (2023): 1544–1554, 10.1093/jnci/djad169. 37603716 PMC10699798

[2] H. Damgacioglu , K. Sonawane , Y. Zhu , et al., “Oropharyngeal Cancer Incidence and Mortality Trends in all 50 States in the US, 2001‐2017,” JAMA Otolaryngology. Head & Neck Surgery 148, no. 2 (2022): 155, 10.1001/jamaoto.2021.3567. 34913945 PMC8678903

[3] A. Costantino , J. S. Magnuson , E. M. Graboyes , U. Alamoudi , and B. H. Haughey , “Temporal and Geographic Trends in HPV Testing for Oropharyngeal Cancer in the United States,” Otolaryngology and Head and Neck Surgery 173, no. 1 (2025): 134–143, 10.1002/ohn.1259. 40211672

[4] E. A. Mroz and J. W. Rocco , “MATH, a Novel Measure of Intratumor Genetic Heterogeneity, Is High in Poor‐Outcome Classes of Head and Neck Squamous Cell Carcinoma,” Oral Oncology 49, no. 3 (2013): 211–215, 10.1016/J.ORALONCOLOGY.2012.09.007. 23079694 PMC3570658

[5] S. C. Cantrell , B. W. Peck , G. Li , Q. Wei , E. M. Sturgis , and L. E. Ginsberg , “Differences in Imaging Characteristics of HPV‐Positive and HPV‐Negative Oropharyngeal Cancers: A Blinded Matched‐Pair Analysis,” American Journal of Neuroradiology 34, no. 10 (2013): 2005–2009, 10.3174/ajnr.A3524. 23660291 PMC3951375

[6] S. Tian , J. M. Switchenko , J. Jhaveri , et al., “Survival Outcomes by High‐Risk Human Papillomavirus Status in Nonoropharyngeal Head and Neck Squamous Cell Carcinomas: A Propensity‐Scored Analysis of the National Cancer Data Base,” Cancer 125, no. 16 (2019): 2782–2793, 10.1002/cncr.32115. 31012957 PMC6663628

[7] K. K. Ang , J. Harris , R. Wheeler , et al., “Human Papillomavirus and Survival of Patients With Oropharyngeal Cancer,” New England Journal of Medicine 363, no. 1 (2010): 24–35, 10.1056/nejmoa0912217. 20530316 PMC2943767

[8] E. A. Mroz , A. M. Tward , R. J. Hammon , Y. Ren , and J. W. Rocco , “Intra‐Tumor Genetic Heterogeneity and Mortality in Head and Neck Cancer: Analysis of Data From the Cancer Genome Atlas,” PLoS Medicine 12, no. 2 (2015): e1001786, 10.1371/journal.pmed.1001786. 25668320 PMC4323109

[9] J. S. Lewis , B. Beadle , J. A. Bishop , et al., “Human Papillomavirus Testing in Head and Neck Carcinomas: Guideline Update,” Archives of Pathology & Laboratory Medicine 149, no. 6 (2025): e115–e150, 10.5858/arpa.2024-0388-CP. 40126379

[10] R. J. Gillies , P. E. Kinahan , and H. Hricak , “Radiomics: Images Are More Than Pictures, They Are Data,” Radiology 278, no. 2 (2016): 563–577, 10.1148/radiol.2015151169. 26579733 PMC4734157

[11] D. Vos , N. Yaffe , C. I. Cabrera , N. M. Fowler , and B. D. D'Anza , “Diagnostic Performance of Radiomics Modeling in Predicting the Human Papillomavirus Status of Oropharyngeal Cancer: A Systematic Review and Meta‐Analysis,” Cureus (2025), 10.7759/cureus.82085. PMC12066096 40351986

[12] P. Krzyszczyk , A. Acevedo , E. J. Davidoff , et al., “The Growing Role of Precision and Personalized Medicine for Cancer Treatment,” Technology (Singap World Sci) 06, no. 03n04 (2018): 79–100, 10.1142/s2339547818300020. PMC6352312 30713991

[13] J. D. Ebben , D. M. Treisman , M. Zorniak , R. G. Kutty , P. A. Clark , and J. S. Kuo , “The Cancer Stem Cell Paradigm: A New Understanding of Tumor Development and Treatment,” Expert Opinion on Therapeutic Targets 14, no. 6 (2010): 621–632, 10.1517/14712598.2010.485186. 20426697 PMC3657606

[14] L. Gay , A. M. Baker , and T. A. Graham , “Tumour Cell Heterogeneity,” F1000Research 5 (2016), 10.12688/f1000research.7210.1. PMC4776671 26973786

[15] S. Elrefaey , M. A. Massaro , S. Chiocca , F. Chiesa , and M. Ansarin , “HPV in Oropharyngeal Cancer: The Basics to Know in Clinical Practice,” Acta Otorhinolaryngologica Italica 34, no. 5 (2014): 299–309. 25709145 PMC4299160

[16] R. J. Gillies and Y. Balagurunathan , “Perfusion MR Imaging of Breast Cancer: Insights Using “Habitat Imaging.”,” Radiology 288, no. 1 (2018), 10.1148/RADIOL.2018180271. 29714676

[17] E. Sala , E. Mema , Y. Himoto , et al., “Unravelling Tumour Heterogeneity Using Next‐Generation Imaging: Radiomics, Radiogenomics, and Habitat Imaging,” Clinical Radiology 72, no. 1 (2017): 3–10, 10.1016/j.crad.2016.09.013. 27742105 PMC5503113

[18] J. Wu , M. F. Gensheimer , N. Zhang , et al., “Tumor Subregion Evolution‐Based Imaging Features to Assess Early Response and Predict Prognosis in Oropharyngeal Cancer,” Journal of Nuclear Medicine 61, no. 3 (2020): 327–336, 10.2967/JNUMED.119.230037. 31420498 PMC7067523

[19] H. Elhalawani , A. L. White , J. Zafereo , et al., “Radiomics Outcome Prediction in Oropharyngeal Cancer,” Cancer Imaging Archive (2018), 10.7937/tcia.2020.2vx6-fy46.

[20] Matched Computed Tomography Segmentation and Demographic Data for Oropharyngeal Cancer Radiomics Challenges,” Scientific Data 4 (2017), 10.1038/sdata.2017.77. PMC5497772 28675381

[21] K. Clark , B. Vendt , K. Smith , et al., “The Cancer Imaging Archive (TCIA): Maintaining and Operating a Public Information Repository,” Journal of Digital Imaging 26, no. 6 (2013): 1045–1057, 10.1007/s10278-013-9622-7. 23884657 PMC3824915

[22] H. Xu , W. Lv , H. Feng , et al., “Subregional Radiomics Analysis of PET/CT Imaging With Intratumor Partitioning: Application to Prognosis for Nasopharyngeal Carcinoma,” Molecular Imaging and Biology 22, no. 5 (2020): 1414–1426, 10.1007/s11307-019-01439-x. 31659574

[23] S. J. Sujit , M. Aminu , T. V. Karpinets , et al., “Enhancing NSCLC Recurrence Prediction With PET/CT Habitat Imaging, ctDNA, and Integrative Radiogenomics‐Blood Insights,” Nature Communications 15, no. 1 (2024): 3152, 10.1038/s41467-024-47512-0. PMC11009351 38605064

[24] R. Achanta , A. Shaji , K. Smith , A. Lucchi , P. Fua , and S. Süsstrunk , “SLIC Superpixels Compared to State‐Of‐The‐Art Superpixel Methods,” IEEE Transactions on Pattern Analysis and Machine Intelligence 34, no. 11 (2012): 2274–2282, 10.1109/TPAMI.2012.120. 22641706

[25] X. Huang , X. Huang , Y. Xie , et al., “Integrative Habitat Analysis and Multi‐Instance Deep Learning for Predictive Model of PD‐1/PD‐L1 Immunotherapy Efficacy in NSCLC Patients: A Dual‐Center Retrospective Study,” BMC Medical Imaging 25, no. 1 (2025): 288, 10.1186/s12880-025-01828-5. 40676504 PMC12272984

[26] X. Huang , X. Huang , K. Wang , H. Bai , B. Ye , and G. Jin , “Habitat‐Based Radiomics From Contrast‐Enhanced CT and Clinical Data to Predict Lymph Node Metastasis in Clinical N0 Peripheral Lung Adenocarcinoma ≤ 3 Cm,” Scientific Reports 15, no. 1 (2025): 17085, 10.1038/s41598-025-02181-x. 40379768 PMC12084560

[27] T. Calinski and J. Harabasz , “A Dendrite Method for Cluster Analysis,” Communications in Statistics ‐ Theory and Methods 3, no. 1 (1974): 1–27, 10.1080/03610927408827101.

[28] P. Whybra , A. Zwanenburg , V. Andrearczyk , et al., “The Image Biomarker Standardization Initiative: Standardized Convolutional Filters for Reproducible Radiomics and Enhanced Clinical Insights,” Radiology 310, no. 2 (2024): e231319, 10.1148/radiol.231319. 38319168 PMC10902595

[29] N. Lunardon , G. Menardi , and N. Torelli , “ROSE: A Package for Binary Imbalanced Learning,”.

[30] S. M. Lundberg , G. Erion , H. Chen , et al., “From Local Explanations to Global Understanding With Explainable AI for Trees,” Nature Machine Intelligence 2, no. 1 (2020): 56–67, 10.1038/s42256-019-0138-9. PMC7326367 32607472

[31] M. Lin , J. F. Wynne , B. Zhou , et al., “Artificial Intelligence in Tumor Subregion Analysis Based on Medical Imaging: A Review,” Journal of Applied Clinical Medical Physics 22, no. 7 (2021): 10–26, 10.1002/acm2.13321. 34164913 PMC8292694

[32] J. Wu , H. Meng , L. Zhou , et al., “Habitat Radiomics and Deep Learning Fusion Nomogram to Predict EGFR Mutation Status in Stage I Non‐Small Cell Lung Cancer: A Multicenter Study,” Scientific Reports 14, no. 1 (2024): 15877, 10.1038/s41598-024-66751-1. 38982267 PMC11233600

[33] S. Wang , T. Wang , S. Guo , et al., “Whole Tumour‐ and Subregion‐Based Radiomics of Contrast‐Enhanced Mammography in Differentiating HER2 Expression Status of Invasive Breast Cancers: A Double‐Centre Pilot Study,” British Journal of Cancer 131 (2024), 10.1038/s41416-024-02871-9. PMC11554679 39379571

[34] Y. Dong , S. Yang , X. Jing , et al., “Habitat Imaging Radiomics Increases the Accuracy of a Nomogram for Predicting Ki‐67‐Positivity in Laryngeal Squamous Cell Carcinoma,” European Journal of Radiology Open 14 (2025): 100659, 10.1016/j.ejro.2025.100659. 40487595 PMC12145539

[35] I. El Naqa , P. W. Grigsby , A. Apte , et al., “Exploring Feature‐Based Approaches in PET Images for Predicting Cancer Treatment Outcomes,” Pattern Recognition 42, no. 6 (2009): 1162–1171, 10.1016/j.patcog.2008.08.011. 20161266 PMC2701316

[36] P. A. Boot , S. W. Mes , C. M. de Bloeme , et al., “Magnetic Resonance Imaging Based Radiomics Prediction of Human Papillomavirus Infection Status and Overall Survival in Oropharyngeal Squamous Cell Carcinoma,” Oral Oncology 137 (2023): 106307, 10.1016/j.oraloncology.2023.106307. 36657208

[37] H. Bagher Ebadian , F. Siddiqui , A. Ghanem , et al., “Radiomics Outperforms Clinical Factors in Characterizing Human Papilloma Virus (HPV) for Patients With Oropharyngeal Squamous Cell Carcinomas,” Biomedical Physics & Engineering Express (2021), 10.1088/2057-1976/ac39ab. 34781281

[38] Y. M. Park , J. Lim , Y. W. Koh , S. Kim , and E. C. Choi , “Machine Learning and Magnetic Resonance Imaging Radiomics for Predicting Human Papilloma Virus Status and Prognostic Factors in Oropharyngeal Squamous Cell Carcinoma,” Head & Neck 44, no. 4 (2022): 897–903, 10.1002/hed.26979. 35044020

[39] O. Altinok and A. Guvenis , “Interpretable Radiomics Method for Predicting Human Papillomavirus Status in Oropharyngeal Cancer Using Bayesian Networks,” Physica Medica 114 (2023): 102671, 10.1016/j.ejmp.2023.102671. 37708571

[40] T. Meeradevi , S. Sasikala , L. Murali , N. Manikandan , and K. Ramaswamy , “Lung Cancer Detection With Machine Learning Classifiers With Multi‐Attribute Decision‐Making System and Deep Learning Model,” Scientific Reports 15, no. 1 (2025): 8565, 10.1038/s41598-025-88188-w. 40075131 PMC11903677

[41] G. Thibault , J. Angulo , and F. Meyer , “Advanced Statistical Matrices for Texture Characterization: Application to Cell Classification,” IEEE Transactions on Biomedical Engineering 61, no. 3 (2014): 630–637, 10.1109/TBME.2013.2284600. 24108747

[42] B. Song , K. Yang , J. Garneau , et al., “Radiomic Features Associated With HPV Status on Pretreatment Computed Tomography in Oropharyngeal Squamous Cell Carcinoma Inform Clinical Prognosis,” Frontiers in Oncology 11 (2021), 10.3389/fonc.2021.744250. PMC8454409 34557418

[43] Y. Zhu , A. S. R. Mohamed , S. Y. Lai , et al., “Imaging‐Genomic Study of Head and Neck Squamous Cell Carcinoma: Associations Between Radiomic Phenotypes and Genomic Mechanisms via Integration of the Cancer Genome Atlas and the Cancer Imaging Archive,” JCO Clinical Cancer Informatics 3 (2019): 1–9, 10.1200/CCI.18.00073. PMC6874020 30730765

[44] S. V. Puram , M. Mints , A. Pal , et al., “Cellular States Are Coupled to Genomic and Viral Heterogeneity in HPV‐Related Oropharyngeal Carcinoma,” Nature Genetics 55, no. 4 (2023): 640–650, 10.1038/s41588-023-01357-3. 37012457 PMC10191634

[45] D. J. Peace , E. Izumchenko , and D. Sidransky , “Unexpected Heterogeneity in Oropharyngeal Squamous Cell Tumors,” Nature Genetics 55, no. 4 (2023): 534–535, 10.1038/s41588-023-01360-8. 37016098

[46] T. de Perrot , V. Lenoir , M. Domingo Ayllón , N. Dulguerov , M. Pusztaszeri , and M. Becker , “Apparent Diffusion Coefficient Histograms of Human Papillomavirus–Positive and Human Papillomavirus–Negative Head and Neck Squamous Cell Carcinoma: Assessment of Tumor Heterogeneity and Comparison With Histopathology,” American Journal of Neuroradiology 38, no. 11 (2017): 2153–2160, 10.3174/ajnr.A5370. 28912282 PMC7963577

[47] H. Mehanna , M. Taberna , C. von Buchwald , et al., “Prognostic Implications of p16 and HPV Discordance in Oropharyngeal Cancer (HNCIG‐EPIC‐OPC): A Multicentre, Multinational, Individual Patient Data Analysis,” Lancet Oncology 24, no. 3 (2023): 239–251, 10.1016/S1470-2045(23)00013-X. 36796393

