# Supplementary material for: Habitat Radiomics Predict HPV Status in Oropharyngeal Cancer
Source: Cancer Med. 2025 Dec 21;14(24):e71481. doi: 10.1002/cam4.71481 (PMC12718549; doi:10.1002/cam4.71481)
Supplement: Supplementary file 1 — Data S1: Supplementary Figures and Tables [file CAM4-14-e71481-s001.docx]

**Supplementary Data**

**1. Mathematical Details of Two-Level Clustering for Habitat Generation**

This section provides additional technical details and mathematical formulation of the two-level clustering pipeline used to generate tumor habitats from radiomic features.

**1. 1. Individual-Level Clustering (Supervoxel Generation)**

At the individual (patient) level, each primary Gross Tumor Volume (GTVp) region was first processed to extract a fused image combining normalized CT intensities and entropy-filtered CT values. The fused image was computed using voxel-wise addition:

Fused Image=CT_ROI_map​+_CT_LocalEntropy_map_

The tumor region on in the fused image was then oversegmented using the SLIC (Simple Linear Iterative Clustering) algorithm. The number of supervoxels was adjusted according to tumor size. This produced a supervoxel label map of the tumor. The algorithm was implemented in MATLAB 2024b with a compactness value of 0.01.

The SLIC algorithm segments an image into superpixels (or supervoxels in 3D) by grouping voxels with similar intensity and spatial proximity. The distance measure D combines color (intensity) distance and spatial distance as follows:

$$D=\sqrt{\left( \frac{d_{c}}{m} \right)^{2}+\left( \frac{d_{s}}{S} \right)^{2}}$$

where:

- *d_c_* is is the Euclidean distance in intensity space
- *d_s_* is the Euclidean spatial distance in voxel coordinates.
- *S* is approximate size of each supervoxel (grid interval)
- *m* is compactness parameter controlling the balance between intensity and spatial proximity

*Radiomics Feature Extraction from Each Supervoxel*

For each supervoxel, 20 first-order statistical features were extracted using intensity histograms from both CT and entropy images. These included: skewness, kurtosis, mean, median, first quartile, third quartile, interquartile range, standard deviation, variance, and energy (10 from each modality × 2 = 20 total). Thus, each supervoxel was represented as a 20-dimensional feature vector.

**1.2. Population-Level Clustering (Habitat Generation)**

The K-means algorithm was employed to analyze the multidimensional feature space. All supervoxel-level feature vectors from all patients were pooled to form a population-level dataset. After extracting supervoxel features from all patients, the K-means algorithm was applied to group similar supervoxels into common habitats across the population. The K-means algorithm was implemented in MATLAB 2024b. The performance of the clustering was evaluated using the Calinski–Harabasz Index. Cluster numbers from 3 to 10 were tested to determine the optimal value. The CH Index is calculated as:

$$CH\mathrm{index}=\frac{SSB}{SSW} x \frac{(N - K)}{(K - 1)}$$

where:

- SSB : Between-cluster sum of squares
- SSW : Within-cluster sum of squares
- N : Total number of samples
- K : Number of clusters

K-means is an unsupervised learning method that partitions data into K distinct clusters by iteratively updating the cluster centroids to minimize the within-cluster sum of squares. The objective function (J) minimized by K-means is:

$$J=\sum_{i=1}^{N} \sum_{k=1}^{K} w_{ik}\times\parallel x_{i}-\mu_{k}\parallel^{2}$$

where:

- *J* is the total clustering cost (also called the within-cluster sum of squared distances).
- *N* is the number of data points (supervoxels).
- *K* is the number of clusters (habitats).
- w_ik_ is a indicator variable (1 if data point *i* belongs to cluster *k*, otherwise 0)
- x_i_ is feature vector of data point *i*.
- μ_k_ is the centroid of cluster *k*.
- ∥x_i_-μ_k_ ∥^2^ is the squared Euclidean distance between data point *i* and centroid *k*.

**2.Habitat Feature Aggregation Strategies**

To determine which habitat-level feature set to use in the habitat radiomics classifier, we tested several feature aggregation strategies, including: maximum feature value, largest volume, sum, mean, minimum, and variance. These strategies were designed to capture different aspects of the tumor subregions identified through habitat analysis. Among them, selecting features from the habitat with the maximum feature value yielded the best classification performance and was therefore used in the final habitat radiomics classifier. A detailed comparison of these strategies is provided in Table S1.

All classifiers were trained using a Support Vector Machine (SVM) with a radial basis function (RBF) kernel. Each classifier underwent its own nested cross-validation framework to ensure fair and unbiased evaluation. A 7-fold nested cross-validation was used to tune hyperparameters and assess performance. In each fold, the model was trained using the selected features, and the optimal hyperparameters were identified through 3-fold cross-validation within the training data, using the area under the ROC curve (AUC) as the selection metric. The trained model was then evaluated on the corresponding held-out test set. After completing all folds, the model from the fold with the highest test AUC was selected as the final classifier.

| **Table S1. Performance comparison of different Habitat Aggregation Strategies** | | | | | |
| --- | --- | --- | --- | --- | --- |
| Habitat Feature Aggregation Strategies | Training AUC | Test AUC | Test Accuracy | Test Sensitivity | Test Specificity |
| Maximum Feature Value | **0.97** | **0,937** | **0,821** | **0,792** | 1 |
| Largest Volume Feature Value | 0.939 | 0,889 | 0,667 | 0,625 | 1 |
| Sum Feature Value | 0.919 | 0,847 | 0,556 | 0,5 | 1 |
| Mean Feature Value | 0.828 | 0,823 | 0,5 | 0,583 | 0 |
| Minimum Feature Value | 0.809 | 0,812 | 0,679 | 0,667 | 0,75 |
| Variance Feature Value | 1 | 0,781 | 0,714 | 0,667 | 1 |

**3. Performance Evaluation of All Classifiers for the Training and Test Datasets**

Table S2 presents the classification performance of three radiomics models: the Habitat Radiomics Classifier, Intratumoral Radiomics Classifier, and Combined Radiomics Classifier, evaluated on both training and test datasets. Reported metrics include AUC, accuracy, sensitivity, specificity, precision, recall, and F1-score for both positive and negative classes. All metrics were calculated using the best-performing outer fold selected during nested cross-validation. The Habitat Radiomics Classifier was developed using features aggregated from the habitat with the maximum feature value, which yielded the best predictive performance among the tested aggregation strategies (see Table S1).

| **Table S2. Performance Evaluation of All Classifiers for the Training and Test Datasets** | | | | | | | | | | | | | |
| --- | --- | --- | --- | --- | --- | --- | --- | --- | --- | --- | --- | --- | --- |
| **Datasets** | Radiomics  Classifier | AUC | Accuracy | Sensitivity | Specificity | Precision (positive class) | Precision (Negative class) | Recall (positive class) | Recall (negative class) | | F1 (positive class) | F1 (negative class) |  |
| **Training** | Habitat | 0.970 (0.942 - 0.997) | 0.908 (0.846 - 0.946) | 0.956 (0.747 - 0.922) | 0.855 (0.878 - 0.985) | 0.878 (0.747 - 0.922) | 0.956 (0.878 - 0.985) | 0.956 (0.854 - 0.982) | 0.855 (0.785 - 0.935) | 0.915 | | 0.903 |  |
|  | Combined | 0.938 (0.897 - 0.978) | 0.862 (0.792 - 0.911) | 0.897 (0.808 - 0.960) | 0.823 (0.750 - 0.914) | 0.847 (0.710 - 0.898) | 0.897 (0.802 - 0.949) | 0.897 (0.771 - 0.940) | 0.823 (0.747 - 0.912) | 0.871 | | 0.858 |  |
|  | Intratumor | 0.897 (0.842 - 0.978) | 0.832 (0.759 - 0.886) | 0.759 (0.700 - 0.874) | 0.890 (0.798 - 0.960) | 0.846 (0.798 - 0.943) | 0.759 (0.635 - 0.850) | 0.759 (0.724 - 0.891) | 0.890 (0.725 - 0.920) | 0.800 | | 0.819 |  |
| **Test** | Habitat | 0.937 (0.843-1.00) | 0.821 (0.644 - 0.921) | 0.792 (0.595 - 0.908) | 1.000 (0.701 - 1.000) | 1.000 (0.595 - 0.908) | 1.000 (0.510 - 1.000) | 0.792 (0.832 - 1.000) | 1.000 (0.189 - 0.733) | 0.884 | | 1.000 |  |
|  | Combined | 0.854 (0.671 - 1.000) | 0.821 (0.644 - 0.921) | 0.875 (0.698 - 0.963) | 0.500 (0.376 - 0.964) | 0.913 (0.690 - 0.957) | 0.500 (0.150 - 0.850) | 0.875 (0.732 - 0.976) | 0.500 (0.118 - 0.769) | 0.894 | | 0.500 |  |
|  | Intratumor | 0.806 (0.578 -1.00) | 0.704 (0.515 - 0.841) | 0.708 (0.494 - 0.877) | 0.667 (0.607 - 0.990) | 0.944 (0.508 - 0.851) | 0.667 (0.208 - 0.939) | 0.708 (0.742 - 0.990) | 0.667 (0.063 - 0.547) | 0.810 | | 0.667 |  |

**4. Habitat, Intratumoral and Combined Radiomics Features**

Table S3 lists the radiomic features selected for each classifier: the Habitat Radiomics Classifier, Intratumoral Radiomics Classifier, and Combined Radiomics Classifier. Feature selection was performed using LASSO logistic regression based on training data in the best-performing outer fold. For the habitat model, features were aggregated using the maximum feature value strategy across tumor subregions. The combined classifier includes the union of features selected in both the habitat and intratumoral models.

| **Table S3. Radiomics Feature Sets Used in Each Classifier** | |
| --- | --- |
| **Classifier** | **Radiomic Features** |
| Habitat Radiomics Classifier | Habitat_Compactness2_3D  Habitat_MajorAxisLength3D  Habitat_Flatness3D  Habitat_VolumeDensityConvexHull3D  Habitat_AngularSecondMomentAveraged3D  Habitat_InformationCorrelation1Merged3D  Habitat_SmallDistanceEmphasis3D  Habitat_LowDependenceLowGrayLevelEmphasis3D |
| Intratumoral Radiomics Classifier | Intratumoral_Compactness2_3D  Intratumoral_MajorAxisLength3D  Intratumoral_Flatness3D  Intratumoral_VolumeDensityConvexHull3D  Intratumoral_InformationCorrelation2Averaged3D  Intratumoral_NormalisedInverseDifferenceMomentMerged3D  Intratumoral_NormalisedGrayLevelNonUniformity3D  Intratumoral_ZoneSizeEntropy3D  Intratumoral_LowDependenceLowGrayLevelEmphasis3D  Intratumoral_DependenceCountEnergy3D |
| Combined Radiomics Classifier | Habitat_Compactness2_3D  Habitat_MajorAxisLength3D  Habitat_Flatness3D  Habitat_VolumeDensityConvexHull3D  Habitat_AngularSecondMomentAveraged3D  Habitat_InformationCorrelation1Merged3D  Habitat_SmallDistanceEmphasis3D  Habitat_LowDependenceLowGrayLevelEmphasis3D  Intratumoral_Compactness2_3D  Intratumoral_MajorAxisLength3D  Intratumoral_Flatness3D  Intratumoral_VolumeDensityConvexHull3D  Intratumoral_InformationCorrelation2Averaged3D  Intratumoral_NormalisedInverseDifferenceMomentMerged3D  Intratumoral_NormalisedGrayLevelNonUniformity3D  Intratumoral_ZoneSizeEntropy3D  Intratumoral_LowDependenceLowGrayLevelEmphasis3D  Intratumoral_DependenceCountEnergy3D |

**5. LASSO Feature Selection for Intratumoral Radiomics Classifier**


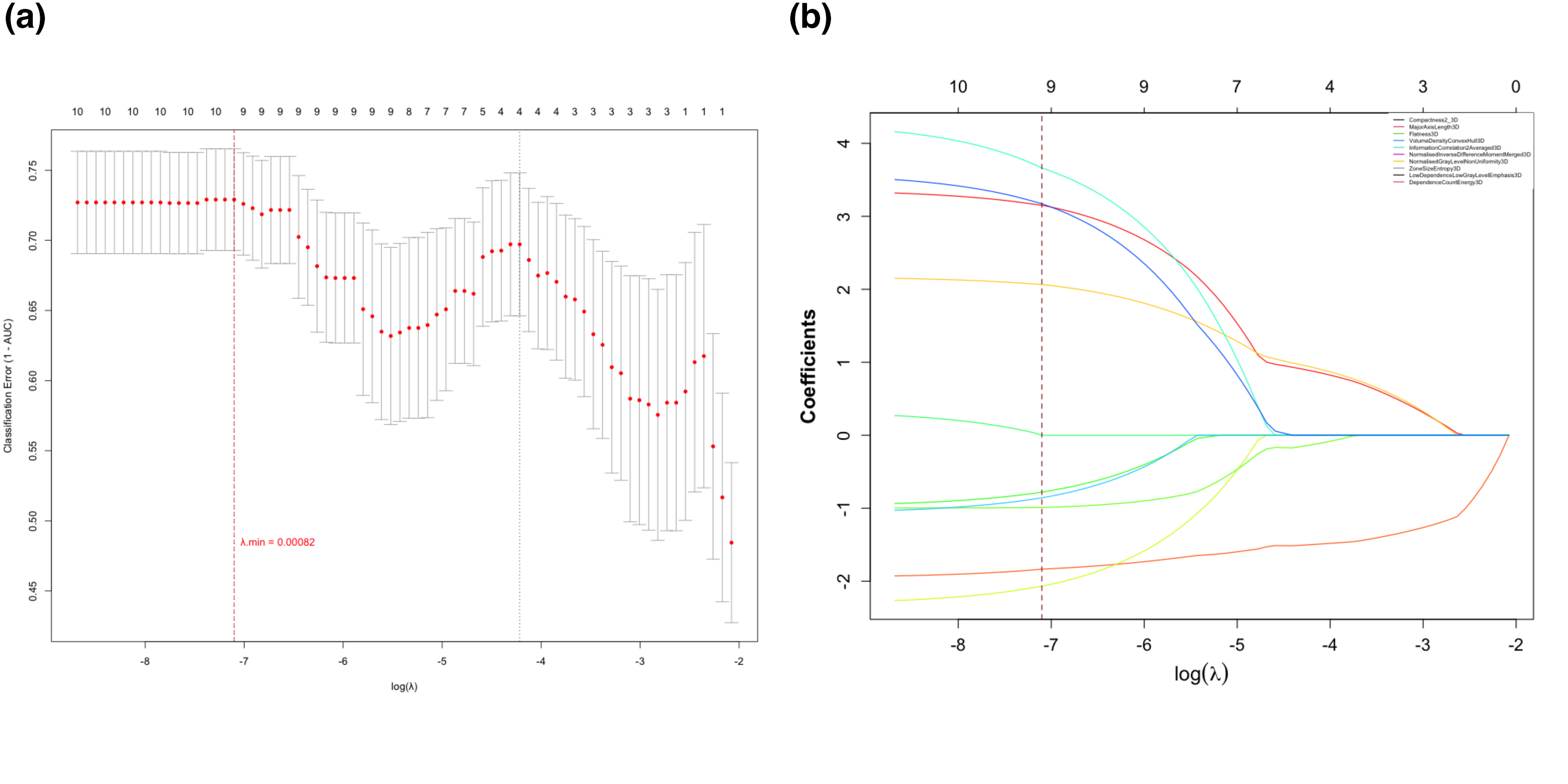


**Figure S1 Feature selection for the Intratumoral Radiomics Classifier using LASSO logistic regression.** (a) Tuning parameter (λ) selection in the LASSO. Classification error (1 – AUC) was plotted against log(λ). The optimal value of λ was selected using 10-fold cross-validation and is indicated by the vertical red dashed line. (b) LASSO coefficient profiles of the features. Each colored line represents the trajectory of a specific feature's coefficient. The vertical red dashed line marks the selected λ value, where 10 features had nonzero coefficients.

**6. SHAP beeswarm plot for the Intratumoral Radiomics Classifier**


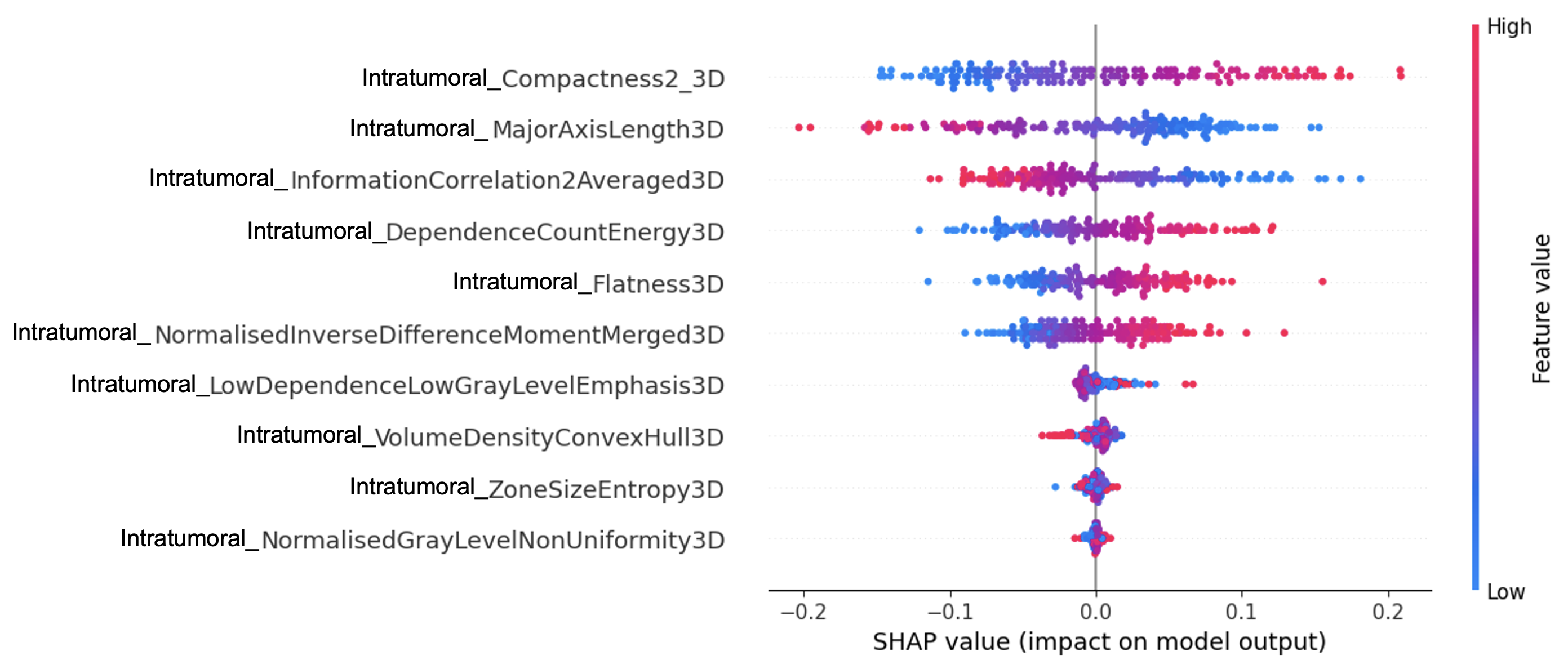


**Figure S2. SHAP beeswarm plot for the Intratumoral Radiomics Classifier, illustrating the impact of selected features on HPV status prediction in the training dataset.** Each point represents a patient, with the SHAP value on the x-axis indicating the contribution of a specific feature to the classifier’s output. Positive SHAP values correspond to a higher likelihood of predicting HPV-positive status, while negative values indicate a tendency toward HPV-negative predictions. The color of each point reflects the actual feature value: red indicates higher feature values and blue indicates lower feature values.

**7. Box Plot Visualization of Discriminative Intratumoral Features**

Figure S3 illustrates the distribution of the radiomic features selected for the Intratumoral Radiomics Classifier, comparing HPV-positive and HPV-negative patients to provide complementary insights into feature behavior.


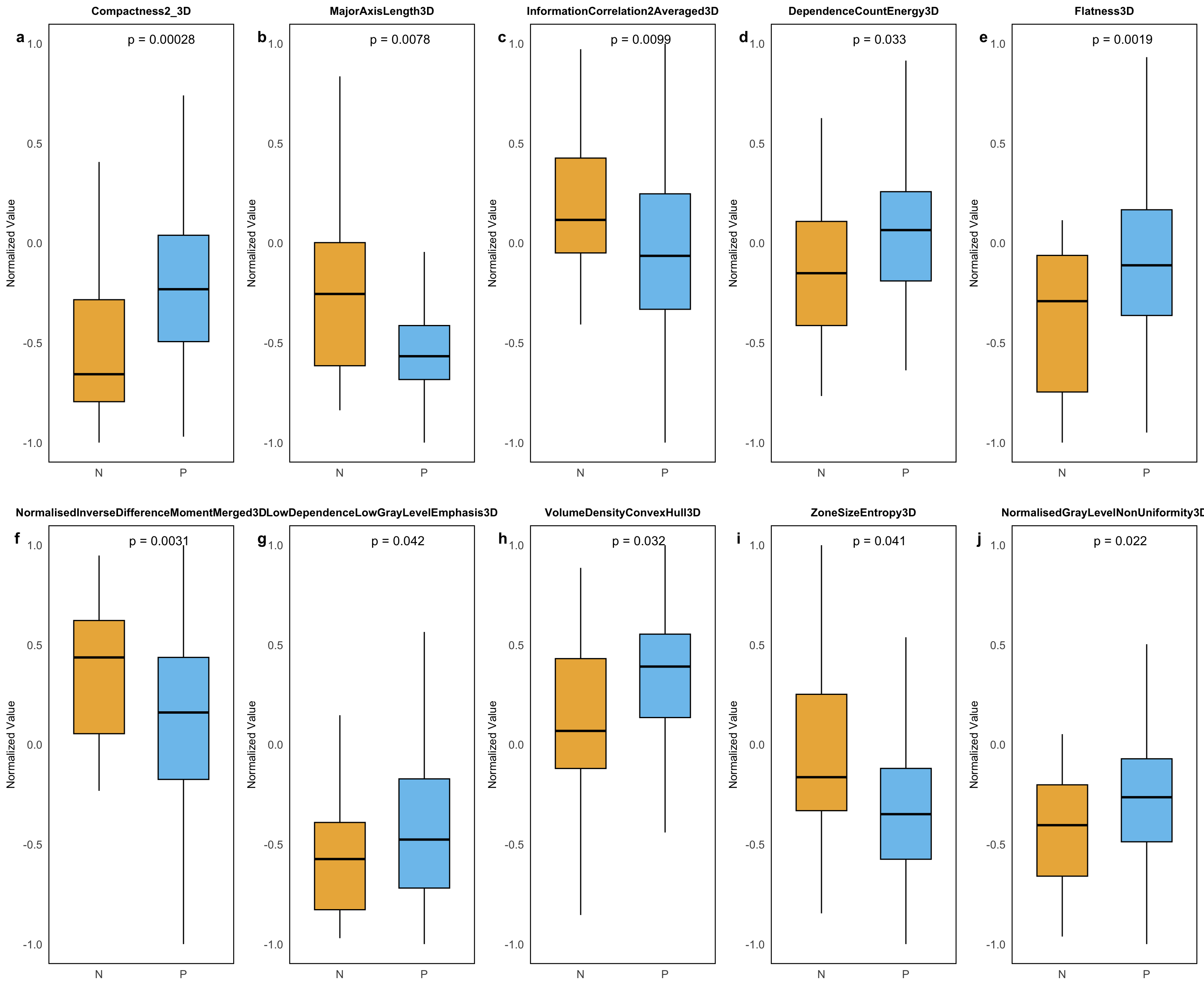


**Figure S3.** **Box plots of radiomic features selected for the Intratumoral Radiomics Classifier.**

| **Table S4. Follow-up time (years) by HPV status (IHC vs HRC).** | | |
| --- | --- | --- |
| **Group** | **N** | **Follow-up Time (years), Median [IQR]** |
| IHC HPV+ | 166 | 6.34 [5, 7.76] |
| IHC HPV- | 26 | 5.33 [1.95, 7.13] |
| HRC HPV+ (predicted) | 167 | 6.26 [4.91, 7.81] |
| HRC HP V- (predicted) | 25 | 6.17 [5.22, 7.06] |
